# Novel multi-epitope vaccine candidate for lumpy skin disease: Computational design and recombinant expression

**DOI:** 10.14202/vetworld.2025.2273-2286

**Published:** 2025-08-09

**Authors:** Aman Kumar, Kamlesh Kumar, Savita Budania, Kamlesh Kumari, Pawan Kumar, Sushila Maan, Kanisht Batra, Narender K. Dhania

**Affiliations:** 1 Department of Animal Biotechnology, Lala Lajpat Rai University of Veterinary and Animal Sciences, Hisar, Haryana, India; 2 Department of Chemistry, Kirori Mal College, University of Delhi, New Delhi, India; 3 Department of Zoology, Faculty of Science, University of Delhi, New Delhi, India

**Keywords:** *Escherichia coli*, immunoinformatics, lumpy skin disease virus, molecular docking, multi-epitope vaccine, recombinant protein expression, toll-like receptors

## Abstract

**Background and Aim:**

Lumpy skin disease (LSD) is a severe transboundary viral infection in cattle, caused by the LSD virus (LSDV), leading to economic losses in the livestock industry. Conventional live-attenuated vaccines face limitations such as strain recombination, incomplete protection, and adverse effects. Therefore, safer and more targeted vaccine strategies are urgently needed. This study aimed to design, simulate, and express a novel multi-epitope vaccine (MEV) candidate against LSDV using a computational immunoinformatic pipeline.

**Materials and Methods:**

Four immunogenic LSDV proteins – P35, A4L, A33R, and L1R – were selected based on their structural and antigenic significance. B- and T-cell epitopes were predicted and filtered using antigenicity, allergenicity, and toxicity criteria. Selected epitopes were linked using specific linkers and an adjuvant to construct an MEV. Molecular docking was performed with bovine toll-like receptors (TLRs), and stability was evaluated through molecular dynamic simulations (GROMACS and iMODS). Codon optimization and heterologous expression of the construct were performed in *Escherichia coli* using the pET-28a(+) vector. Expression was checked through sodium dodecyl sulfate-polyacrylamide gel electrophoresis (SDS-PAGE) and Western blot.

**Results:**

A total of 23 epitopes from the four LSDV proteins were incorporated into a 514 amino acid-long vaccine construct. The designed construct demonstrated high antigenicity, non-allergenicity, solubility, and favorable physicochemical properties. Docking with bovine TLR4 revealed stable binding with significant interaction residues. Molecular dynamics confirmed structural stability over 50 ns simulations. The recombinant construct was successfully expressed as a ~59 kDa His-tagged protein in *E. coli*, confirmed by SDS-PAGE and Western blotting.

**Conclusion:**

This study demonstrates a comprehensive computational and experimental workflow for developing a multi-epitope subunit vaccine against LSDV. The MEV candidate shows strong immunogenic potential, structural stability, and recombinant expression feasibility, offering a promising alternative to traditional vaccines. Further *in vivo* evaluation is warranted to assess protective efficacy.

## INTRODUCTION

Originally confined to the Saharan region of Africa, lumpy skin disease (LSD) has now emerged as a transboundary animal disease. Its presence has been documented in multiple countries, including Israel, Russia, Southeast Europe, and numerous Asian nations such as Pakistan, Myanmar, Nepal, Vietnam, Hong Kong, India, Iran, and China [1, 2]. Owing to its substantial threat to the livestock industry, the World Organization for Animal Health has classified LSD as a notifiable disease in bovines. In 2022, a major outbreak occurred across various regions of India, resulting in high morbidity and mortality rates and causing significant economic damage to the dairy industry. While cattle are the primary hosts of LSDV, spontaneous cases have also been observed in water buffaloes. The causative agent, LSDV, is a double-stranded DNA virus belon-ging to the *Poxviridae* family and *Capripoxvirus* genus, with a genome size of approximately 151 kilobase pairs [1, 3]. Its genome is linear and AT-rich, comprising 156 predicted genes, and shares 96% nucleotide similarity with other *Capripoxviruses* [4]. LSDV exhibits a close genetic relationship with sheep pox and goat pox viruses, making serological differentiation among them challenging [5].

Transmission of LSD primarily occurs through insect vectors, especially biting flies and mosqui-toes [6]. Although less common, direct transmission through contact with infected animals is also possible. In vector-borne transmission, insects infect susceptible animals, and ticks may also play a role as carriers. Direct transmission can occur through exposure to infe-cted secretions such as saliva, skin lesions, respiratory discharges, milk, and semen. Notably, the disease can spread over distances >100 km and rapidly affect entire herds. In severe outbreaks, morbidity rates can reach up to 85%, and the absence of effective control measures can result in significant mortality [7].

Preventive vaccination with live-attenuated *Capripoxvirus* strains is the current strategy for LSD control [5]. However, vaccine failures have been reported, primarily due to issues such as inadequate strain characterization, host specificity, and safety con-cerns. Since 2014, Turkey has experienced recurring LSD outbreaks despite regular heterologous vaccination, indicating that these vaccines may not offer sufficient protection [8].

Despite the availability of live-attenuated vaccines against LSD virus (LSDV), their use is increasi-ngly challenged due to safety concerns, limited cross-protection, and the risk of recombination with circulating field strains, potentially leading to vaccine-derived outbreaks and novel virulent variants [5, 8]. In addition, the immunological basis of protection remains incompletely understood, particularly regarding the specific epitopes responsible for eliciting a robust and protective immune response. Although recent studies have proposed multi-epitope vaccine (MEV) constructs using individual proteins such as core or membrane glycoproteins [3, 9, 10], these approaches have not fully leveraged the combined immunogenic potential of proteins from both the intracellular mature virus (IMV) and extracellular enveloped virus (EEV) forms. Furthermore, limited studies have extended their *in silico* findings to experimental validation through recombinant expression, which is critical for downstream application in vaccine development. Thus, there is a pressing need for a rationally designed, experimentally validated, multi-antigenic epitope-based vaccine candidate that is safer, broadly immunogenic, and suitable for recombinant expression in heterologous systems.

This study aims to develop a novel, rationally designed MEV candidate targeting LSDV by integrating conserved B-cell and T-cell epitopes from four immunodominant proteins: P35, A4L, A33R, and L1R. Using a comprehensive immunoinformatic pipeline, the selected epitopes were evaluated for antigen-icity, allergenicity, and toxicity and were systematically assembled using appropriate linkers and adjuvants. The designed construct was subjected to molecular docking with bovine toll-like receptors (TLRs) and further validated through molecular dynamic simulations to assess its binding stability. Codon optimization was performed for *Escherichia coli* expression, and the construct was successfully expressed in a recombinant system. This integrative approach seeks to offer a promising and safer alternative to existing vaccines by advancing a computationally engineered and experimentally evalu-ated subunit vaccine candidate against LSDV, paving the way for further *in vivo* evaluation and development.

## MATERIALS AND METHODS

### Ethical approval

The authors did not perform any experiments on animals so ethical approval was not necessary for this study.

### Study period and location

The study was conducted from December 2023 to December 2024 at the Department of Animal Biotechnology, Lala Lajpat Rai University of Veterinary and Animal Sciences (LUVAS), Hisar, Haryana, India.

### Retrieval of sequence data and workflow design

**Figure 1:**
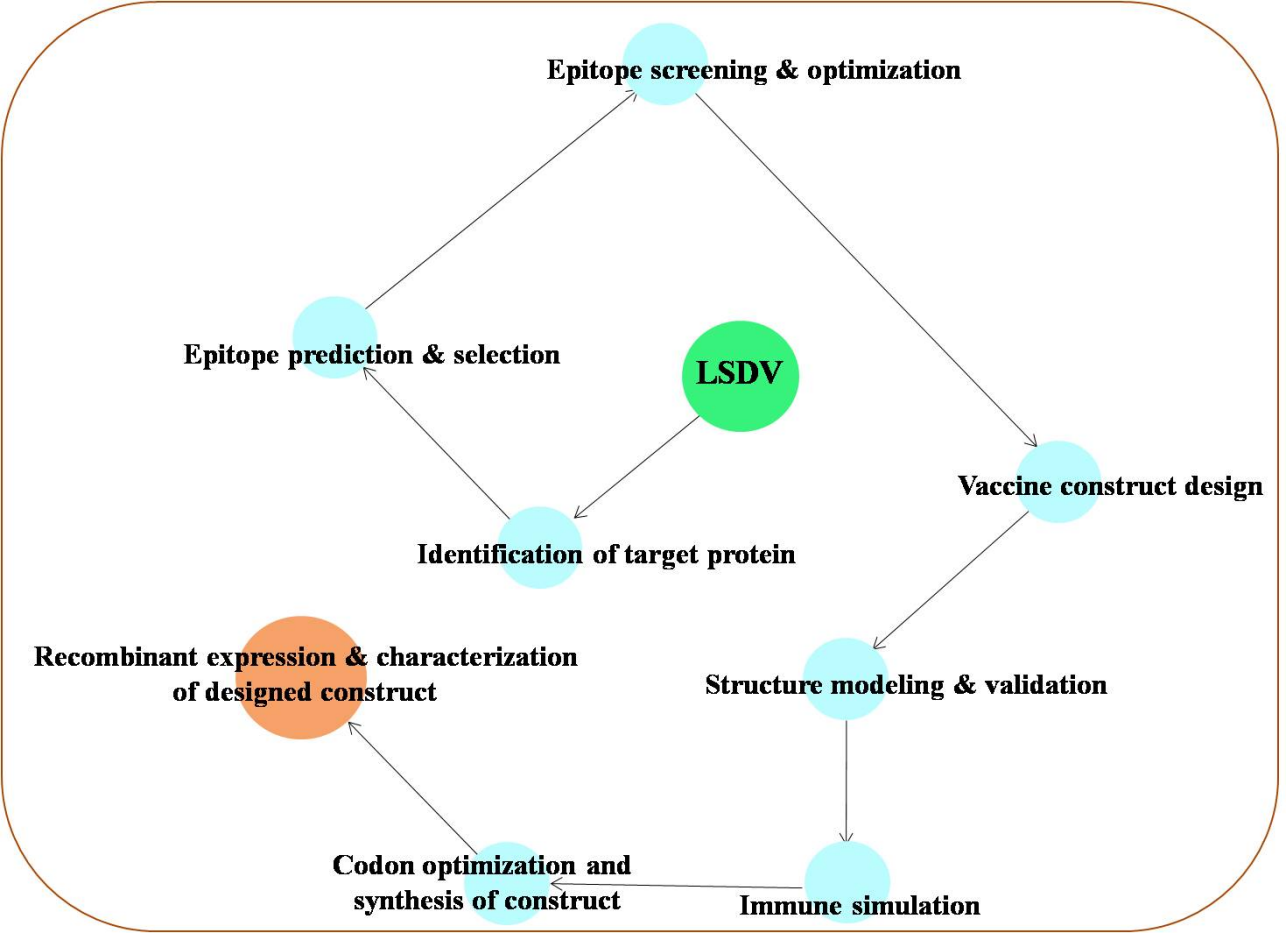
Graphical flowchart for designing a candidate multi-epitope vaccine for lumpy skin disease virus.

All the nucleic acid and targeted protein sequences used in the study for bioinformatics and immunoinformatics were retrieved from the National Center for Biotechnology Information (NCBI) and the protein database. The graphical flow chart in Figure 1 represents the development process of the MEV can-didate, delineating the important steps involved in the methodology.

### Selection of target proteins

Four LSDV proteins – P35, A4L, A33R, and L1R – were selected for epitope prediction based on their structural and functional importance in LSDV infection and immunogenicity. P35, a major membrane pro-tein, plays a key role in virion architecture and is highly immunogenic [11]. A4L is also an immunodominant protein with a high antigenic index [12]. A33R, a conserved type II membrane glycoprotein, facilitates EEV formation and long-range viral spread, making it a promising vaccine candidate [13]. L1R is essential for membrane fusion and viral entry, and its potential as a recombinant subunit vaccine has been reported [14]. The respective sequences of the selected proteins were obtained from the NCBI and UniProt databases (https://www.ncbi.nlm.nih.gov; https://www.uniprot.org/). The homologous sequences of antigenic proteins of LSD-NI 2490 strains were identified using NCBI BlastP [15]. NCBI BLAST tools were used to obtain non-redundant sequences and were aligned using BioEdit 7.0 to identify conserved regions (http://www.mbio.ncsu.edu/). Sequences with accession numbers AAK85035.1, AAK85056.1, and AAK85021.1 were retrieved and used in the study.

### Epitope prediction for B and T lymphocytes

The selected proteins (Table 1) were subjected to both linear and discontinuous epitope predictions. B lymphocyte prediction was conducted using various servers to minimize the chance of oversight. The sequential B-cell epitope predictors, BepiPred version 2.0 (threshold value: 0.50) and ABCpred version 1.0 (threshold value: 0.51; 65.93% accuracy with equal sensitivity and specificity), were used to detect epitopes and non-epitope fragments (http://tools.immuneepitope.org/, https://webs.iiitd.edu.in/) [16, 17]. In addition, tri-peptide similarity and propensity score-based immune epitope database-dependent vector tools were used for B-cell antigenic epitope prediction (http://sysbio.unl.edu/) [18]. Linear and discontinuous epitopes from the three-dimensional (3D) protein structure were predicted using ElliPro version 3.0 (parameters: minimum score 0.5, maximum score 6 Å) (http://tools.iedb.org/) [19]. Prediction of proteasomal cleavage for T lymphocyte epitopes (NetChop/NetCTL/NetCTLpan, method C-term 3) was performed using a neural network architecture-based approach, with a threshold set at 0.9 (http://tools.iedb.org/). This comprehensive prediction encompassed major histocompatibility complex (MHC) binding, C-terminal cleavage affinity, and antigen transporter efficiency [20]. Protein sequences were also analyzed for naturally processed MHC II ligands, predicting probability scores based on length and cleavage motifs at the N- and C-termini (http://tools.iedb.org/mhciinp/) [21].

**Table 1:** List of the selected proteins and their sequences.

S. No.	Protein	Amino acid sequences
1	AAK85035.1/LSD-NI2490[LSDV074/P35]-IMV	MADIPLYVIPIVGREISDVVPELKSDNDIFYKKVDTVKDFKNSDVNFFFKDKKDISLSYKFLIWEKVEKSGGVENFTEYFSGLCNALCTKEAKSSIAKHFSLW KSYADADIKNSENKFIVVIEDDNTLKDLITIHNIIIEMQEKNIDIFQLRETFHNSNSRILFNQENNNFMYSYTGGYDFTLSAYVIRLSSAIKIINEIIKNKGISTSLSFEMYKLE KELKLNRQVLNDSSKYILHNTKYLSKKRANEMKNGIWNRVGKWMAHRFPDFSYYVSHPLVSFFGIFDISIIGALIILFIIIMIIFDLNSKLLWFLAGMLFTYII
2	AAK85056.1/LSD/NI-2490[LSDV095/A4L]-Core protein	MDFMKKYTKDLETTVKNKKDEEIASTSNLINNTSVTLTDVDTMLKSKEHLYQQMMMNQLEEKKTLKIKNIEIKNNSNKLNDQCSEKKQNDPLKKIKSI SHDELVKELKDIKDKTKSLQDDSDSLIKDISVAKDTTFDAINSIMNDLKKRFNIDKLDDNNSK
3	AAK85083.1/LSD NI-2490[LSDV122L/A33R]-EEV	MLVDIPKSGTETDYDESNNFTAFAGSTIYGYGLKSKKNIKKKVKLINFCIKISIMASMVSLITITILLAFFNNTCELNQFKEHKPYFLKNPNPTTYSDDDT ESELNVYRSCKGIVYSGYCYTFNSEPKSFNDAYDDCEKKNSELPSNNLMNDWISDYLDGTWGEDGNVLFKEKNQELETIDISDEMRSYYCVRSFF
4	AAK85021.1/LSD NI-2490 [LSDV060/L1R]-IMV	MGAAASIQTTVNTLNEKISSKLEQTAEATAEAKCDIEIGSIVFRQNKGCNVTVKNLCSSKAESQ LDAILKAATETYDSLTPDQKAYVPGLMTAALNIQTSVNTVVKDFETYVKQKCTSKSVIDNKLKIHNIFIDECAAPTGTTTNF EFINSGTSQGICAIKTLMDVTTKASTKFSPSQSSGYGYQFYIIAAVVVILSMVFLYYVKKMLFTSTKDKIKIILANKPEVHWTSYLDTFFSNTPTIIEK

### BoLA allele-based cytotoxic T lymphocytes (CTL) epitope analysis

We used TepiTool in the current analysis to study bovine-specific alleles (http://tools.iedb.org/tepitool/). The IEDB-based computational prediction method handles homologous peptides [22]. In this study, cattle were selected as the host species, and a bovine MHC class I allele was targeted for epitope prediction. Specifically, six bovine leukocyte antigen (BoLA) class I alleles were considered: BoLA-D18.4, BoLA-JSP.1, BoLA-HD6, BoLA-T2a, BoLA-T2b, and BoLA-T2c. Dupl-icate peptides were excluded, and peptides with lengths ranging from 8 to 14 mers were considered. Approximately 1057 peptides were included in the prediction analysis. The NetMHCpan-based prediction method was employed, and peptide selection was based on the predicted half maximal inhibitory concentration (IC_50_), with a cutoff set at 500 nM [23].

### Screening for antigenicity, allergenicity, and toxicity

The predicted epitopes were further screened for antigenicity, allergenicity, and solubility, as it was important to predict protective antigens and suitable subunit vaccines. An alignment-independent, auto cross-covariance transformation of proteins was performed for each predicted epitope using VaxiJen v2.0, threshold: 0.4 (http://www.ddg-pharmfac.net/) [24]. Allergenicity was assessed using cross-covariance transformation-based screening through AllergenFP v1.0 and AllerTOP v2.0 servers (https://ddg-pharmfac.net/) [25].

### Construction of the MEV sequence

The selected epitopes were linked using defined linkers. The “EAAAK” linker was placed between the adjuvant (P02584.2 | Profilin-1) and the epitopes to enhance immunogenicity and improve vaccine stability and functionality. Linkers “AAY”, “GPGPG”, and “KK” were used for CTL, helper T lymphocyte (HTL), and B-cell epitopes, respectively. The positions of the epitopes were selected based on their location in the original protein sequence. A 6× His-tag (“HHHHHH”) was added to the final assembled construct.

### Secondary structure prediction and transmembrane topology

The secondary structure of the protein seq-uence was predicted using two feed-forward neural network tools (http://bioinf.cs.ucl.ac.uk/). The anal-ysis was conducted using PSPIRED v4.0 with strin-gent cross-validation, achieving an accuracy score of 81.6%. MEMSAT3-SVM schematics were used to predict transmembrane topology and helices, achieving >78% accuracy. Fold recognition was performed using GenTHREADER (http://bioinf.cs.ucl.ac.uk/psipred/), which calculated template length, coverage, solvation, alignment, and pairwise potentials.

### Solubility and physicochemical properties

The screened epitopes were evaluated for solubility using the Protein-Sol server (https://protein-sol.manchester.ac.uk/) [26], which provided theoretical predictions of solubility, feature deviation, fold propensity, and net segment charge. Results were presented in graphical and text format. Various chemical and physical parameters were computed using ToxinPred and ProtParam tools (https://web.expasy.org/protparam/, https://webs.iiitd.edu.in/) [27], including pI, atomic composition, positive and negative residues, extinction coefficients, aliphatic index, and hydropathicity.

### Tertiary structure modeling and refinement

ColabFold, employing AlphaFold2 through MMseqs2, was used to model the protein struc-ture (https://github.com/sokrypton/ColabFold) [28]. MMseqs2 (UniRef + Environmental) was applied in unpaired + paired mode. The predicted structure was refined using GalaxyRefine server, which rebuilds side chains and relaxes the structure through molecular sim-ulations [29]. The refined model was evaluated based on root mean square deviation (RMSD), global dista-nce test-high accuracy (GDT-HA), MolProbity scores, stereo-chemical quality, and Ramachandran plot analy-sis (https://prosa.services.came.sbg.ac.at/and https://saves.mbi.ucla.edu/) [30].

### Molecular docking with TLR4

The vaccine protein was analyzed for its inter-action with TLR4. Due to the unavailability of the bovine TLR4 crystal structure, the human TLR4 (D299G and T399I) structure was used (https://www.rcsb.org/structure/4g8a.1.B) [31]. A template model was created through ProMod3 using target-template alignment [27]. Model quality was evaluated using global model quality estimation, distance distribution, QMEAN Z-score, and Ramachandran plot analysis.

The refined MEV candidate and modeled bovine TLR4 structure were subjected to docking. Interaction analysis was performed using Hex 8.0.0 with parameters set to “potential to N = 25 (5525 coefficients) using 12 Tasks, Grid: 236 × 236 × 236 = 13,144,256 cells (1,307,412 non-zero) of 0.60 Å,” integrated over 1,307,412 cells in 9.08 s (144,020/s) using DARS potential and a spherical polar Fourier correlations algorithm. In addition, ClusPro (https://cluspro.bu.edu/home.php) was also used for docking. The ClusPro server removed unstructured regions, evaluated pairwise distances, and incorporated small-angle X-ray scattering data to identify binding sites [32]. Multiple low-energy models were generated, and the best-scoring complex was selected. Docked interactions were visualized using LigPlot+ v2.2 and BIOVIA Discovery Studio (https://www.ebi.ac.uk/thornton-srv/software/LigPlus/).

### Molecular dynamics and normal mode analysis

Stability and structural conformation of the vaccine-TLR complex were assessed through dihe-dral coordinate analysis using GROMACS and iMODS (https://imods.iqfr.csic.es/). Before GROMACS simulation, both the vaccine and TLR were solvated and neutralized with hydrogen ions and counter ions. Energy minimization and MD simulation were run with the following parameters: Time step 1 femtosecond, simulation length 50 nanoseconds (ns), temperature 300 K, and pressure 1 atmosphere. Post-simulation analyses included RMSD, radius of gyration (Rg), and trajectory evaluations. iMODS was used to perform normal mode analysis, including Cα atoms, 100 interface modes, edNMA elastic network model, fixed angle ratio, and inflection point evaluation. Structural stability was characterized by deformability plots, B-factor values, eigenvalues, and covariance matrix [33].

### Codon optimization and *in vitro* expression

The codon sequence was optimized using the JCAT tool to improve translational efficiency (http://www.jcat.de/). Optimization involved parameters, such as guanine-cytosine (GC) content, cytosine–phosphate–guanine islands, splicing sites, cis-regulatory elements, stop codons, mRNA structure, RNA stability, restriction sites, and elimination of unwanted motifs. The His-tagged codon-optimized sequence was synthesized by Biolinkk India Pvt. Ltd. and cloned into the pET-28a(+) expression vector. Ligation was confirmed through sequencing. The recombinant plasmid was transformed into *E. coli* (BL21) and cultured for 16 h in Luria–Bertani (LB) broth containing kanamycin. Following transformation, both qualitative and quantitative analyses were performed to confirm successful cloning. Polymerase chain reaction (PCR) amplification was carried out using T7 universal and construct-specific primers. For protein expression, a 5 mL overnight culture harboring the recombinant plasmid was inoculated into 200 mL of LB medium (supplemented with 25 μg/mL kanamycin) at 37°C and 170 rpm. Expression was induced using 1 mM isopropyl β-D-1-thiogalactopyranoside. The expressed multi-epitope protein was subjected to 12% sodium dodecyl sulfate-polyacrylamide (SDS-PAGE) gel electrophoresis and transferred to a polyvinylidene fluoride membrane. Blocking was performed using 5% bovine serum albumin at 37°C for 1 h, followed by washing with PBST (0.05% Tween 20). The membrane was incubated with HRP-conjugated mouse anti-His-tag antibody at 37°C for 1 h under continuous shaking. Excess antibody was removed by washing, and 3,3′-diaminobenzidine was used as the chromogenic substrate for detection. The reaction was stopped by rinsing the membrane with distilled water.

## RESULTS

### Antigenic proteins and sequence retrieval

Various proteins, including extracellular envelope protein (EEV), IMV, and core protein, have been identified as immunogenic proteins of LSDV [34, 35]. To enhance the spectrum of antigens for improved protection, we selected four immunogenic proteins: P35/LSDV074 and L1R of IMV, A33R/LSDV122L of EEV, and A4L of the core protein. The sequences of these selected proteins were retrieved from the NCBI and UniProt databases. Sequences with access-ion numbers AAK85035.1/LSD-NI2490[LSDV074/P35], AAK85056.1/LSD/NI-2490[LSDV095/A4L], AAK85083.1/LSD NI-2490[LSDV122L/A33R], and AAK85021.1/LSD NI-2490[LSDV060/L1R] were further selected for analysis (Table 1). Subsequently, these sequences were used for the analysis of conserved domains.

### B lymphocyte epitope prediction

**Figure 2:**
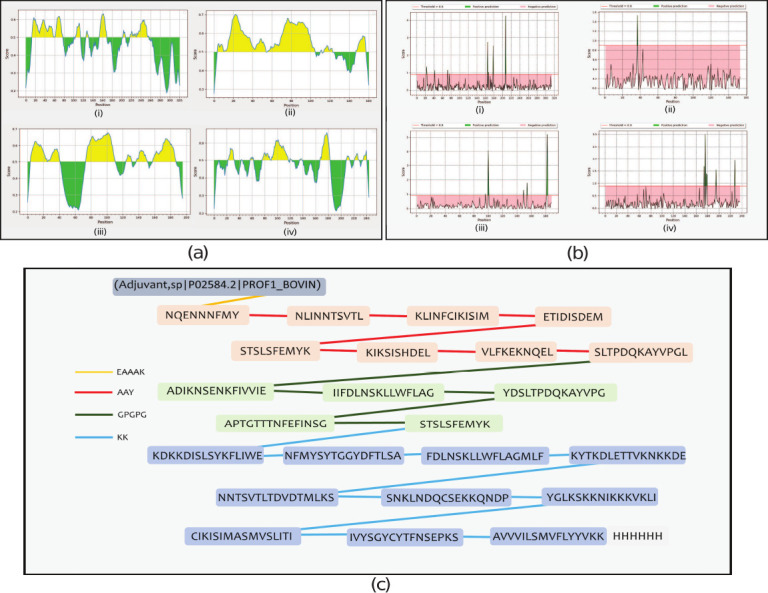
Epitope prediction (a) predicted location of B-cell epitope for four selected immunogenic proteins, i.e., (i) LSDV074/P35, (ii) LSDV095/A4L, (iii) LSDV122L, (iv) LSDV060/L1R. (b) Predicted position of cytotoxic T lymphocytes (CTL) epitopes in four selected immunogenic proteins, i.e., (i) LSDV074/P35, (ii) LSDV095/A4L, (iii) LSDV122L, (iv) LSDV060/L1R. (c) Schematic representations of the multi-epitope vaccine illustrating the incorporation of CTL, helper T lymphocyte, and B-cell epitopes in the designed construct. These epitopes are interconnected using EAAAK (yellow color coded), AAY (red color coded), GPGPG (green color coded), and KK (blue color coded) linkers. LSDV=Lumpy skin disease virus.

Using the random forest algorithm, 8, 5, 6, and 13 vaccine subunits were predicted in LSDV074/P35, LSDV095/A4L, LSDU+0056122L, and LSDV060/L1R, respectively (Figure 2a[i-iv] and Supplementary Table 1). In addition, 27, 17, 16, and 25 B-cell epitope sequ-ences were predicted using the ABCpred server (Supplementary File 1; Data 1). A support vector machine-based B-cell antigenic epitope predictor identified 13 epitopes (Supplementary File 1; Data 2). Among the 130 B-cell epitopes predicted, only 11 were selected to assemble the MEV candidate. Peptides were discarded if they were found to be non-antigenic, with an overall prediction threshold set at the protective antigen threshold value of 0.4. Predicted B-cell epitopes were excluded from further consideration if they were identified as potential allergens during allergenicity analysis and were discarded if they were found to be unsuitable for allergen analysis.

### T lymphocyte epitope prediction

NetCTL predicted 9, 1, 5, and 6 vaccine subunits in LSDV074/P35, LSDV095/A4L, LSDV122L/A33R, and LSDV060/L1R, respectively, as CTL epitopes (Figure 2b[i-iv] and Supplementary Table 2). These sequences were screened for naturally processed MHC II ligands, and based on cleavage probability, the top 5 sequences were selected from each group (Supplementary Table 3). Using bovine-specific alleles and NetMHCpan-based predictions, 45, 28, 18, and 27 vaccine subunits were identified in LSDV074/P35, LSDV095/A4L, LSDV122L/A33R, and LSDV060/L1R, respectively, as Class-I MHC epitopes (Supplementary Table 4). The final list was based on an IC_50_ set at 500 nM. T-cell epitopes were screened for toxicity and allergens, and unsuitable epitopes were discarded. The selected and screened epitopes are listed in Figure 2c.

### MEV assembly and secondary structural features

**Figure 3:**
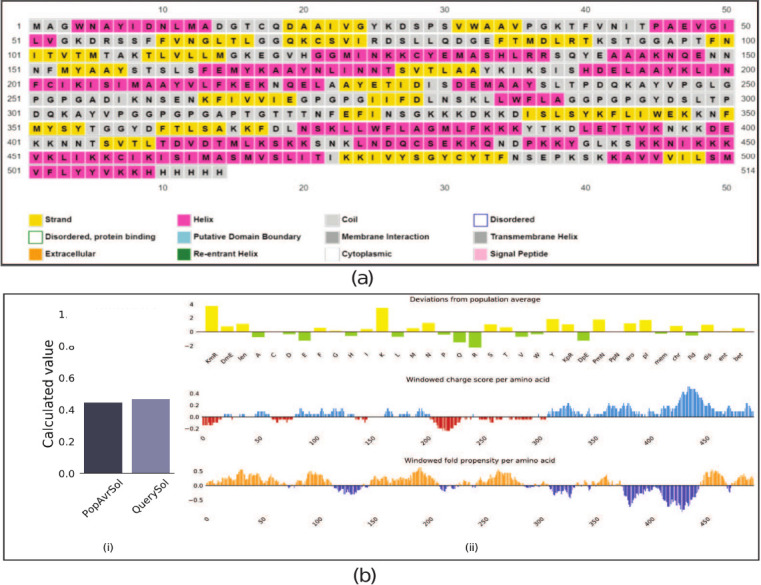
Topology and solubility. (a) Secondary structure prediction schematics for transmembrane topology and helix, highlighting the structural features of the vaccine. (b) Predicted scaled solubility for the assembled sequence, (i) solubility (protein-sol calculation), (ii) windowed charge and fold propensity score of an assembled construct.

The MEV was designed by incorporating epitopes predicted by high-scoring B and T lymphocytes, as depicted in Figure 2c. The sequence was constructed by arranging adjuvants, CTL epitopes, HTL epitopes, and B-cell epitopes and was terminated with a 6× His-tag to facilitate protein identification and purification. The immune properties of the sequence were reinforced by introducing EAAAK, AAY, GPGPG, and KK linkers sequentially (Figure 2c). Secondary structure features were predicted, and analysis revealed the presence of helix, coil, and transmembrane configurations; notably, no disordered fragments were observed (Figure 3a). The transmembrane topology prediction indicated that amino acids 196–211, 460–477, and 493–508 in the sequence represented the pore-lining helix, with the adjuvant part predominantly character-izing the extracellular domain (1–196) (Figure 3a). pGenTHREADER alignment for the same was forecasted using 19 templates, with a net score exceeding 50 and a p-value less than 0 (Supplementary Table 5).

### Vaccine solubility, physicochemical, and toxicological analyses

The observed scaled soluble value was 0.467, with a pI of 9.680 (Figure 3b[i and ii]). The analysis also included the observation of window fold propensity per amino acid (Figure 3b[i and ii]). The estimated protein half-life was predicted to be ~30 h, >20 h, and >10 h for mammalian reticulocytes, yeast, and *E. coli*, respectively. Additional stability parameters, including instability (22.72), aliphatic (79.90), and average of hydropathicity (−0.291), indicated that the protein was stable. Furthermore, the assembled protein was assessed using the ToxinPred server, and no toxin-related features were identified in the epitopes or protein fragments of the sequence (Supplementary Table 6).

### 3D modeling and refinement of vaccine candidate

**Figure 4:**
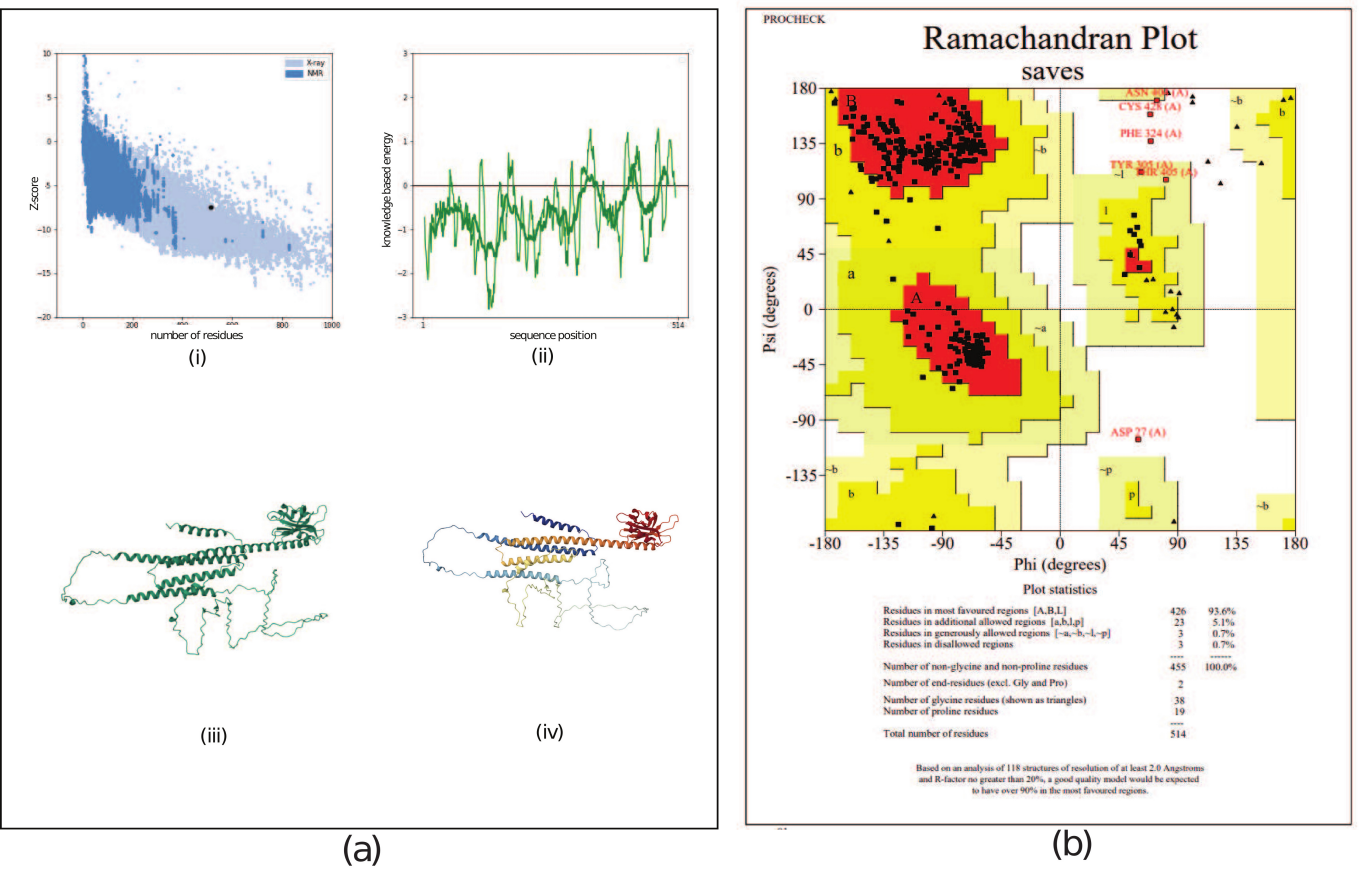
Predicted structure quality score (a) three-dimensional representation of the multi-epitope vaccine, (i) overall model quality, (ii) knowledge-based energy range, (iii) predicted structure conformation, (iv) refined structure using MD simulation. (b) Ramachandran plot analysis of the predicted refined structure.

**Figure 5:**
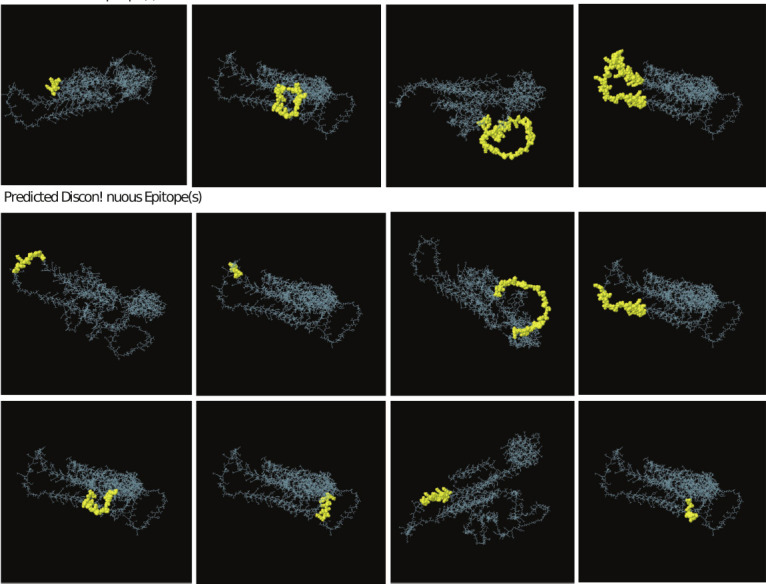
Linear and discontinuous epitope prediction from the three-dimensional modeled multi-epitope vaccine protein.

ColabFold: AlphaFold2 was used to design the 3D structural models. The initial structure demonstr-ated an overall model quality with a z score of –7.54 (Figures 4a and 4i), and local model quality was observed within the KBE range (Figures 4a and 4ii). In terms of the predicted structural conformation, the Chi1-Chi2 plot, bond length, and bond angle properties fell within the favorable region, as determined through an analysis of 163 structures at a resolution of 2.0 (Figure 4b). The predicted structure (Figure 4a[iii]) underwent refinement using molecular dynamic simulation, and repeated structure perturbation was executed for overall structure relaxation with the GalaxyRefine server (Figure 4a[iv]). This refinement led to improvements in the Global Distance Test-HA (0.8818), RMSD (0.666), MolProbity (1.342), and clash score (3.4), as well as the elimination of poor rotamers (reduced to 0.0). The enhanced/refined predicted structure was assessed for error values, and an overall quality factor of 95.4407 was recorded, which significantly validates the protein structure. The predicted structure comprised 426 residues, with 93.6% (most favored), 5.1% (additional allowed), and 0.7% (generously allowed and disallowed) regions (Figure 4b). A total of 38 glycine residues (shown as triangles) and 19 proline residues were observed (Figure 4b). Linear and discontinuous epitope prediction was performed from MEV structure to confirm the antigenic nature of the modeled structure (Figure 5).

### Docking of bovine TLR and MEVs

**Figure 6:**
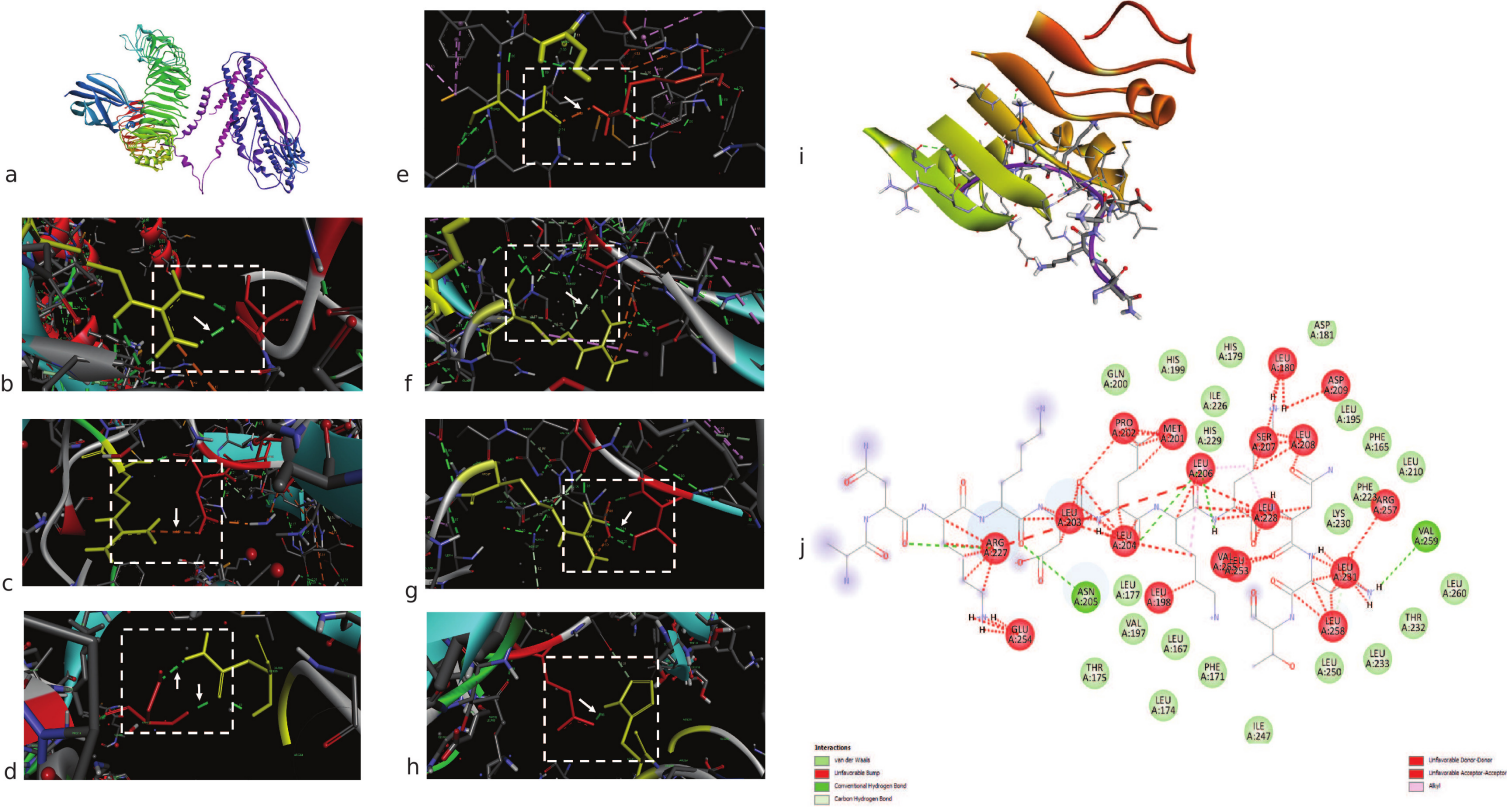
Illustration of protein–protein interactions. (a) Multi-epitope subunit vaccine candidate interaction with bovine toll-like receptor 4 protein. Interacting pocket atoms represent (b) Asp100 (C chain) and Arg234 (A chain), (c) Asp101 (C chain) and Arg264 (A chain), (d) Asn265 (A chain) and Ser103 (C chain), (e) Lys109 (C chain) and Asp60 (A chain), (f) Gly110 (C chain) and Arg87 (A chain), (g) Arg87 (A chain) and Thr112 (C chain), (h) Glu111 (C chain) and His159 (A chain). (i) ribbon representation of interacting pocket atoms. (j) 2D representation of interacting pocket atoms showed carbon-hydrogen bonds, conventional hydrogen bonds, van der Waals bonds, etc.

**Figure 7:**
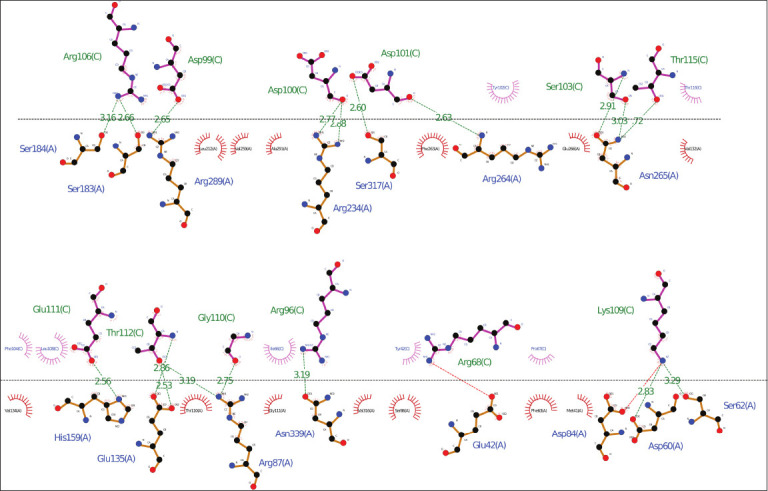
Bovine toll-like receptors 4 and docking and Lig plot analysis of docked protein complex.

For docking analysis, the structures of bovine-specific TLRs were prepared using SWISS-MODEL Homology Modeling (ProMod3 3.2.1). Existing human TLR templates, 4g8a.1.B and 5d3i.1.A, were used for TLR4 (BGD: BT12122; Ensembl: ENSBTAG00000006240) and TLR2 (BGD: BT11801; Ensembl: ENSBTAG00000008008), respectively. However, both structures were constructed in a monomeric state. Model quality estimation revealed significant values for both proteins (GMQE: 0.53, 0.56; QMEAN: 0.75, 0.74). The predicted structures were subjected to docking (ClusPro), and the resulting scores were screened for hydrophobic-favored, electrostatic-favored, and VdW + Elec coefficient scores. The balanced coefficient score-based model cluster 2 was selected for analysis, comprising 30 members, with a −763.4 center-weighted score and a −949.3 lowest score. In another docking analysis (Hex), a correlation summary was acquired by the root mean square (RMS) deviation and steric clashes. Among the 1352 clusters, the top cluster solution showed Etotal (−1496.9), Eshape (−424.1), Eforce (−1072.8), Eair (0.0), Bmp (2), and RMS (−1.00), and the highest negative energy-based compound was selected for further analysis. The TLR4 vaccine compound and docking complex are demonstrated in Figure 6a. The pocket atoms are shown in Figures 6b–h and 2D interaction of these pocket atoms is explained in Figure 6j. Overall, the observations suggest the binding of the predicted MEV construct to the TLR4 protein, with ~17 interactions observed in the LigPlot interaction analysis. The residues involved in the interaction between the TLR4-vaccine complex are illustrated in Figure 7.

### Molecular dynamic simulation

**Figure 8:**
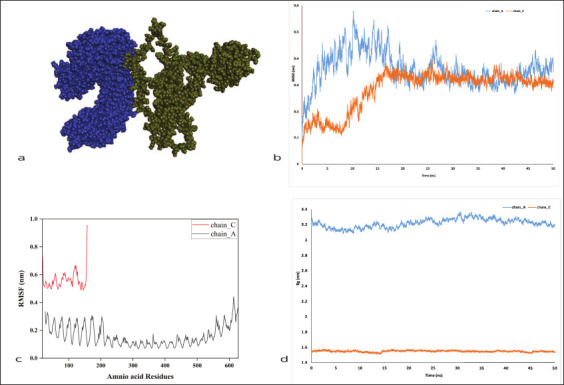
Molecular dynamic simulation study of the construct and toll-like receptors (TLRs)-4, (a) representing the complex protein used for simulation, analysis of (b) root mean square deviation, (c) root-mean-square fluctuation, and (d) radius of gyration of TLR4 and the vaccine complex.

The docked complexes (Figure 8a) were assessed for 50 ns simulations, and post-simulation, structural stability was evaluated using RMSD (Figure 8b). It showed the difference in protein backbone position between start to end of simulation. The root -mean square fluctuation (RMSF) identified stable regions within the complex (Figure 8c), and Rg showed the compactness of the protein (Figure 8d). The RMSD analysis revealed that the TLR4-vaccine complex exhibited consistent stability throughout the 50-ns simulation period (Figure 8b). Specifically, TLR4 chain A: Rg stabilized at approximately 1.6 nm and TLR4 chain B: Rg stabilized at approximately 3.2 nm (Figure 8d). These findings suggest that the docked complex maintained stability with minimal fluctuations during the simulation. Consistent RMSD, RMSF, and Rg values indicate a stable protein-ligand interact-ion. In addition, a molecular dynamic simulation was conducted using the iMODS server. Various parameters were calculated, including protein complex main-chain deformability, regions with high deformability, and experimental B-factor values, all of which indicated satisfactory values for the docked structure internal coordinates (Supplementary File 2). Relative motion stiffness was reported as Eigenvalue (1) = 8.722960e-06, suggesting that a high energy input is needed to deform the structure (Supplementary File 2). The stability was further confirmed through the covariance matrix, variance values, and elastic network model analysis (Supplementary File 2). Overall, the results of the MD simulation confirmed the stability. A similar analysis was performed for other bovine TLRs, and we observed a significant interaction with the MEV (Supplementary File 2).

### Codon optimization and expression

**Figure 9:**
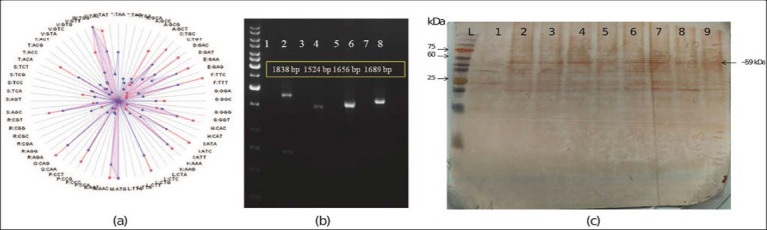
Optimized multi-epitope construct. (a) Relative codon frequency distribution of the optimized multi-epitope, optimized sequence (red), and host (blue). A closer match signifies a more appropriate and optimized codon usage for the host. (b) Electropherogram of PCR amplicons, L: 1 kb ladder, Lane 1, 3, 5, and 7: respective negative control; Lanes 2, 4, 6, and 8: confirmation of recombinant lumpy skin disease virus (rLSDV) construct in the vector using vector-specific primer, construct-specific primer, and combination of both (c) Western blotting. Lane L: molecular weight protein marker. Lane 1, uninduced *Escherichia coli* as a negative control. Lane 2-9, induced *E. coli* having rLSDV protein.

Figure 9a illustrates the comparison of codon usage profiles between the codon-optimized sequence (depicted in red) and the native host sequence (in blue). Several parameters were strategically optimized to enhance the expression efficiency in the heterologous host system. The Codon Adaptation Index (CAI) was increased to 0.91, indicating a high level of compatibility with the host’s preferred codon usage. The GC content was adjusted to 50.19%, which falls within the optimal range for transcription stability. Repetitive sequences and undesirable motifs were eliminated to minimize the risk of transcriptional errors and secondary structure formation. Restricted enzyme recognition sites and negative cis-acting elements were modified or removed to facilitate efficient cloning and transcription. These modifications collectively contributed to improved mRNA stability and an extended predicted half-life, ultimately supporting enhanced protein expression. The PCR products were analyzed by gel electrophoresis on a 1% agarose gel, revealing amplicon sizes of 1838, 1524, 1656, and 1689 bp, respectively (Figure 9b). Sequencing of the recombinant construct also confirmed the presence of the construct in the recombinant plasmid (Supplementary File 3). Furthermore, the presence of recombinant LSDV (rLSDV) and histidine-fused protein was confirmed by western blotting using anti-His tag antibodies (Figure 9c). However, in the negative control, no reactivity was observed (Figure 9c).

## DISCUSSION

### Geographical expansion and outbreak severity of LSD

LSD was historically restricted to African countries; however, several recent outbreaks have been reported in Asian nations and other regions globally. In 2019, India experienced an outbreak characterized by low morbidity and no mortality. In contrast, a severe outbreak in 2022 resulted in the death of approximately 80,000 cattle within 3 months [34]. The affected cattle population in India was found to harbor a distinct LSD genetic lineage. Despite the use of live-attenuated goat pox and sheep pox vaccines, complete protection was not achieved, leading to widespread animal suffering. Furthermore, the administration of live-attenuated vaccines has been linked to the emergence of hybrid viruses with novel transmission properties, potentially leading to new viral strains and increased mortality in cattle [35, 36].

### Limitations of conventional vaccines and need for alternatives

Conventional vaccine development approaches are time-consuming, highlighting the need for alter-native strategies to produce more effective vaccine candidates. In this study, immunogenic proteins of LSDV were selected, and immunodominant epitopes were predicted through *in silico* methods to identify components capable of eliciting a protective immune response against LSD.

### Selection of antigenic proteins for vaccine design

Specifically, the EEV protein A33R, IMV proteins P35 and L1R, and the core protein A4L were identified as antigenic targets. While Kar *et al*. [3] and Shahab *et al*. [9] designed MEV candidates based solely on the core protein, Salauddin *et al*. [10] developed a candidate targeting the membrane glycoproteins of the intracellular enveloped virus and EEV. Given the demonstrated immunogenic properties of EEV (122L/A33R) and IMV (LSDV060 and LSDV074), the inclusion of epitopes from both IMV and EEV proteins is expected to improve the protective immunogenic potential of the vaccine candidate designed in this study [3, 9].

### Antigenicity and host safety evaluation

For antigen prediction, the VaxiJen 2.0 server was employed with a threshold of 0.4, consistent with Shahab *et al*. [9]. The selected proteins were conserved across various LSDV isolates and showed no homology with the host proteome, thereby minimizing the risk of autoimmunity. Considering the importance of B-cell-mediated immunity in viral clearance, B-cell and T-cell epitopes were selected accordingly to construct the MEV candidate.

### Physicochemical features of the designed vaccine

The final designed vaccine construct is 514 amino acids long, with a molecular weight of approximately 59 kDa, and exhibits a favorable solubility score of 0.467 and an isoelectric point (pI) of 9.680. Both the antigenicity index and solubility score exceeded those reported in earlier vaccine designs [3, 9].

### Structural modeling and topological analysis

Secondary structure prediction of the designed candidate revealed no disordered regions. Transmembrane topology analysis indicated three pore-lining helices located at amino acid positions 196–211, 460–477, and 493–508. The adjuvant domain was entirely positioned within the extracellular region, a configuration favorable for eliciting a protective immune response. pGenTHREADER alignment was performed using 19 structural templates, yielding a net score above 50 and a p < 0.

### Tertiary structure validation

The tertiary structure of the MEV candidate showed an overall model quality Z score of −7.54, with local quality measures falling within the knowledge-based energy range, consistent with previously published models by Kar *et al*. [3] and Shahab *et al*. [9].

### Interaction with TLRs

TLRs play a critical role in detecting conserved microbial components. Hence, docking studies were performed to assess interactions between the designed vaccine candidate and TLRs. The docking and molecular simulation results confirmed strong and stable binding.

## CONCLUSION

In this study, a robust MEV candidate agai-nst LSDV was developed using a comprehensive immunoinformatic approach targeting four highly immunogenic LSDV proteins: P35, L1R, A33R, and A4L. The final construct, comprising 514 amino acids with a molecular weight of approximately 59 kDa, demonstrated favorable physicochemical properties, including a high antigenicity score (above the VaxiJen threshold of 0.4), good solubility index (0.467), and a stable (pI = 9.680). Secondary and tertiary structural predictions revealed a well-folded and stable config-uration, free from disordered regions, with three trans-membrane pore-lining helices and a predominantly extracellular adjuvant domain. Tertiary structure refinement yielded high-quality metrics, including a Z-score of −7.54 and a GDT-HA of 0.88, validating the stability and reliability of the model.

Molecular docking and simulation studies with bovine TLR4 revealed a strong and stable interact-ion, characterized by consistent RMSD and Rg values throughout a 50-ns simulation. The eigenvalue from normal mode analysis (8.72e-06) further supported the structural rigidity of the docked complex. Codon optimization improved translational efficiency (CAI: 0.91), and successful expression in *E. coli* BL21 was confirmed by SDS-PAGE and Western blot analysis.

The MEV candidate developed here offers a promising alternative to conventional live-attenuated vaccines, which have demonstrated incomplete prot-ection and a potential role in the emergence of hybrid viruses. This subunit vaccine design offers a safer and more targeted approach with minimal risk of autoimmunity, a feature particularly relevant in regions experiencing high mortality due to evolving LSDV strains.

One of the key strengths of this study lies in the inclusion of both B-cell and T-cell epitopes from multiple immunogenic proteins, which enhances the breadth of immune coverage. The construct underwent comprehensive *in silico* validation for structural integrity, antigenicity, solubility, and safety. Screening for host non-homology further reduces the risk of autoimmune reactions. Codon optimization and expression validation provide additional evidence of translational viability.

However, the study has limitations. Despite extensive computational validation, the immunogenic efficacy and safety profile of the construct must still be confirmed through *in vivo* studies. The docking simulations relied on human TLR templates due to the unavailability of bovine crystal structures, which may introduce minor deviations. Furthermore, the possibility of immunodominance among multiple epitopes could affect the vaccine’s performance in real-world applications.

Future research should focus on evaluating the *in vivo* immunogenicity and protective efficacy of the vaccine in target livestock species. Additional studies are needed to optimize the formulation and adjuvant systems for practical field applications. Cross-protection against various LSDV strains and long-term immune memory assessments will also be critical for advancing this candidate towards deployment.

In conclusion, this study presents a rationally designed, highly stable, and immunologically potent MEV candidate against LSDV. The integration of diverse epitopes, along with robust structural validation and successful expression, highlights its potential as a next-generation vaccine. With further experimental validation and formulation development, this MEV con-struct represents a viable option for effective control and prevention of LSD in cattle.

## DATA AVAILABILITY

The supplementary data can be available from the corresponding author upon a reasonable request.

1. Supplementary file 1:
  - Supplementary data 1: Predicted list of B-cell epitopes using ABCpred.
  - Supplementary data 2: Predicted list of B-cell epitopes using the SVM B-cell antigenic epitope predictor.
2. Supplementary file 2: Molecular dynamic simu-lation analysis of MEV construct and TLR-4 sub-unit, (a) representing relative motion stiffness using eigenvalues and variance, (b) main-chain deformability and B-factor/mobility, (c) covariance matrix, where red color signifies a substantial correlation between residues, and (d) elastic network model matrix. Similarly, bovine TLR2 and TLR9 and epitope vaccine docking.
3. Supplementary file 3: Sequencing result of rLSDV construct
4. Supplementary Table 1: List of BepiPred-2.0 predicted B-cell epitope peptides.
5. Supplementary Table 2: List of NetCTL predicted T-cell epitope peptides.
6. Supplementary Table 3: List of naturally processed MHC II ligands and cleavage probability.
7. Supplementary Table 4: List of predicted Class-I MHC epitopes using bovine-specific alleles.
8. Supplementary Table 5: Predicted pGenTHREADER alignment report for the modelled vaccine candidate.
9. Supplementary Table 6: Toxicity, SVM score, hydro-phobicity, steric hindrance, sidebulk, hydropathicity, amphipathicity, hydrophilicity, and net hydrogen report for the modelled vaccine candidate.

## AUTHORS’ CONTRIBUTIONS

AK: Conceptualization, methodology, formal analysis, writing-original draft, and writing-review and editing. KK: Performed the molecular simulation experiment. KKumari, KB, PK, and SB: Formal analysis, and writing-review and editing. SM: Supervision, and writing-review and editing. NKD: Formal analysis, methodology, writing-original draft, and writing-review and editing. All authors have read and approved the final manuscript.
